# Autoinducer 2-Dependent Escherichia coli Biofilm Formation Is Enhanced in a Dual-Species Coculture

**DOI:** 10.1128/AEM.02638-17

**Published:** 2018-02-14

**Authors:** Leanid Laganenka, Victor Sourjik

**Affiliations:** a Max Planck Institute for Terrestrial Microbiology, Marburg, Germany; b LOEWE Center for Synthetic Microbiology, Marburg, Germany; University of Bayreuth

**Keywords:** autoinducer 2, biofilms, mixed communities, quorum sensing

## Abstract

Biofilms in nature typically consist of multiple species, and microbial interactions are likely to have crucial effects on biofilm development, structure, and functions. The best-understood form of communication within bacterial communities involves the production, release, and detection of signal molecules (autoinducers), known as quorum sensing. Although autoinducers mainly promote intraspecies communication, autoinducer 2 (AI-2) is produced and detected by a variety of bacteria, thus principally allowing interspecies communication. Here we show the importance of AI-2-mediated signaling in the formation of mixed biofilms by Enterococcus faecalis and Escherichia coli. Our results demonstrate that AI-2 produced by E. faecalis promotes collective behaviors of E. coli at lower cell densities, enhancing autoaggregation of E. coli but also leading to chemotaxis-dependent coaggregation between the two species. Finally, we show that formation of such mixed dual-species biofilms increases the stress resistance of both E. coli and E. faecalis.

**IMPORTANCE** The role of interspecies communication in the development of mixed microbial communities is becoming increasingly apparent, but specific examples of such communication remain limited. The universal signal molecule AI-2 is well known to regulate cell-density-dependent phenotypes of many bacterial species but, despite its potential for interspecies communication, the role of AI-2 in the establishment of multispecies communities is not well understood. In this study, we explore AI-2 signaling in a dual-species community containing two bacterial species that naturally cooccur in their mammalian hosts, i.e., Escherichia coli and Enterococcus faecalis. We show that active production of AI-2 by E. faecalis allows E. coli to perform collective behaviors at low cell densities. Additionally, AI-2- and chemotaxis-dependent coaggregation with E. faecalis creates nucleation zones for rapid growth of E. coli microcolonies in mixed biofilms and enhances the stress resistance of both species.

## INTRODUCTION

Living in dense, structured, multicellular communities, such as surface-attached biofilms, generally provides bacteria with a number of fitness advantages, compared to a solitary planktonic lifestyle (1). Bacterial biofilms are present in most ecological niches, including the human body, where they can consist of hundreds of species (2–4). The complexity of multispecies biofilms is accompanied and regulated by a number of interactions within and between species, ranging from cooperation to predation (5–8). The best-understood coordination mechanism of bacterial behavior within a community is cell-density-dependent chemical communication called quorum sensing (QS) (9). QS is based on production of, secretion of, and subsequent concentration-dependent responses to signal molecules (autoinducers). This process plays a role in various types of collective bacterial behaviors, including biofilm formation and also colonization of plant and animal hosts by symbiotic or pathogenic bacteria. An array of different QS molecules and response systems exist, allowing bacteria to establish relationships on both intraspecies and interspecies levels (10).

The best-described broad-range interspecies signaling molecule is autoinducer 2 (AI-2) (11). AI-2 is produced by a range of Gram-positive and Gram-negative bacteria (11, 12) and regulates bioluminescence, biofilm formation, motility, and virulence (13). Although most of these functions have been investigated in communities of individual species, several studies suggested the importance of interspecies communication mediated by AI-2 for establishment of mixed biofilms and development of dental plaque (14–16). Furthermore, AI-2 is also produced by many gut-associated bacteria (17–19), and it was shown to affect the composition of the gut microbiota, favoring Firmicutes while hindering Bacteroides in an antibiotic-treated mouse model (20).

AI-2 is the only known QS molecule produced by the enteric bacterium Escherichia coli, and its production and uptake were shown to affect several E. coli phenotypes, including biofilm formation, motility, and virulence (21, 22). Previous work showed that, during autoaggregation or biofilm formation by E. coli, AI-2 serves as a chemoattractant that recruits planktonic cells to growing cell aggregates (23–25). However, it remained unclear whether and how E. coli could use this AI-2-mediated autoaggregation in mixed microbial communities in which it represents only a minor fraction of the population.

E. coli and the Gram-positive bacterium Enterococcus faecalis both inhabit the human gastrointestinal tract, and they cooccur in catheter-associated urinary tract infections (26, 27). It was shown recently that E. faecalis augments E. coli growth under iron-limited conditions, as found within the host, by secreting l-ornithine, which induces siderophore synthesis in E. coli (28). This suggests that these two species might generally interact in the host during polymicrobial infection. In this study, we describe another level of interaction between E. coli and E. faecalis during the formation of mixed biofilms. We show that AI-2 produced locally by E. faecalis aggregates attracts E. coli cells, leading to enhanced aggregation and microcolony formation by E. coli and to increased stress resistance of both species. Moreover, AI-2 production by E. faecalis allows the E. coli population to maintain the induced state of its QS system, despite low cell densities. Together, these results demonstrate that E. coli can use AI-2 produced by other species to promote its QS-regulated collective behavior at low cell densities. We propose that such interspecies signaling may provide fitness advantages to E. coli or other bacterial species in ecological niches where their relative abundance is low, such as the human gastrointestinal tract.

## RESULTS

### E. faecalis enhances biofilm formation by E. coli.

To investigate possible effects of interspecies communication on biofilm formation, we cocultivated E. coli with E. faecalis in microtiter plates. Such static cultures of E. coli are known to form robust biofilms, in which intercellular interactions are mediated primarily by a major adhesin, antigen 43 (Ag43), at 37°C (as used here) (24, 29, 30) or by curli filaments at 30°C (24, 31, 32).

**FIG 1:**
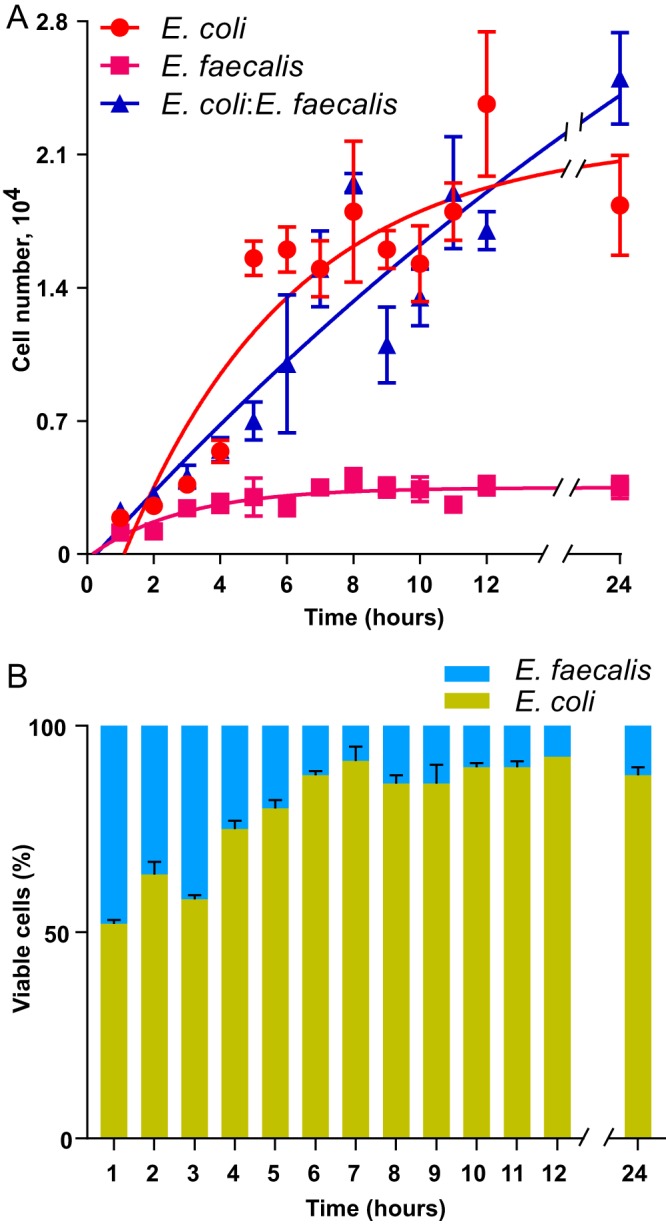
Growth of E. coli and E. faecalis in single- or double-species cultures. (A) Growth rates of static E. coli and E. faecalis single-species cultures (red and magenta dots, respectively) and of mixed E. coli-E. faecalis cultures (blue dots). (B) Composition of static E. coli-E. faecalis biofilm cultures during the first 24 h of incubation. Means of three independent experiments are shown; error bars indicate standard deviations.

**FIG 2:**
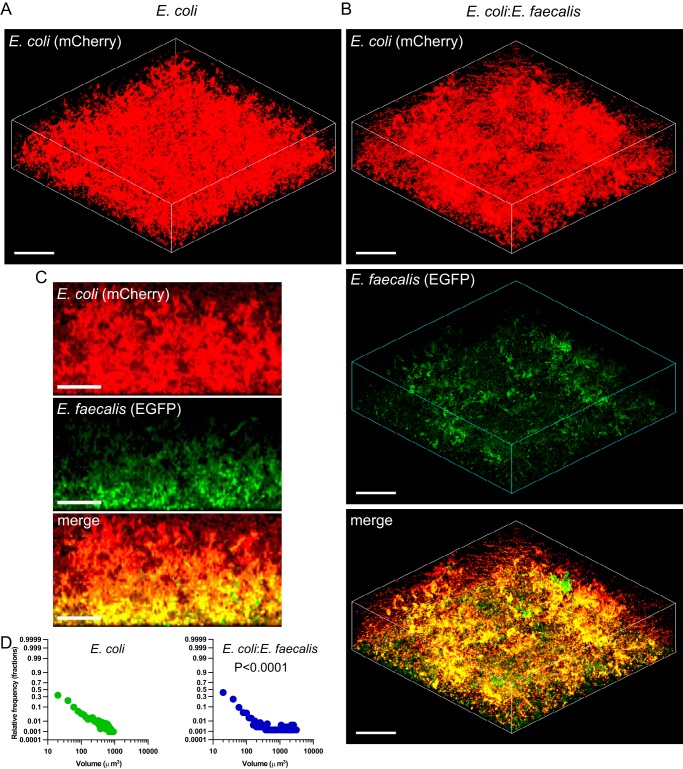
Biofilm formation by E. coli in monocultures or in cocultures with E. faecalis. (A and B) Confocal laser scanning microscopy of static biofilms formed by E. coli (expressing mCherry) grown individually (A) or in a mixed culture with E. faecalis (expressing enhanced GFP [EGFP]) (B). Scale bars, 40 μm. The mixed culture was initially inoculated at 1:1 ratio. (C) Side views of the mixed E. coli-E. faecalis biofilm. Scale bars, 20 μm. (D) Distribution of microcolony volumes in static single- and double-species biofilms of E. coli. The *P* value for the difference between single- and double-species biofilms was calculated using an unpaired *t* test (the data distribution was confirmed to be normal).

We observed that, under these conditions, E. coli cultures reached optical density at 600 nm (OD_600_) values of ∼0.8 at 10 to 12 h postinoculation, whereas the growth of E. faecalis was limited to maximal OD_600_ values of ∼0.15 (Fig. 1A). Consistently, cocultivation of E. coli with E. faecalis had little effect on the overall growth (Fig. 1A), with E. faecalis being quickly overgrown by E. coli and constituting about 10 to 14% of the biofilm biomass after 24 h of cocultivation (Fig. 1B). Nevertheless, we observed that E. coli biofilms formed under these conditions were apparently more structured when grown in cocultures with E. faecalis (Fig. 2A and B; also see Fig. S1A in the supplemental material). Notably, microcolonies formed by E. coli and E. faecalis apparently colocalized within these mixed biofilms (Fig. 2B and C). Image quantification confirmed that E. coli biofilms in mixed communities consisted of significantly larger microcolonies (Fig. 2D). The enhancement of microcolony formation also occurred at a 4-fold-lower E. faecalis inoculum concentration (Fig. S1B and C).

### AI-2 secretion by E. faecalis aggregates attracts chemotactic E. coli cells.

**FIG 3:**
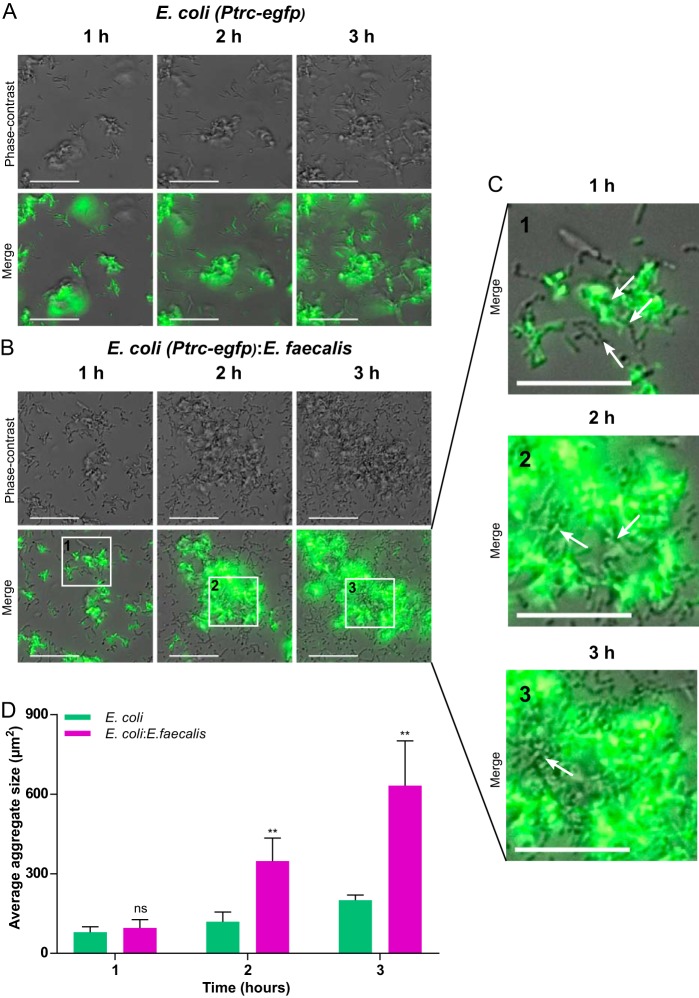
Aggregation of E. coli during early stages of biofilm formation in single- or double-species cultures. (A to C) Aggregates formed at the well surface by E. coli cells (expressing EGFP) grown in monoculture (A) or cocultured with unlabeled E. faecalis (B and C). Cells of E. faecalis can be seen in the phase-contrast channel as distinct chains of round cells or as parts of E. coli-E. faecalis aggregates. Scale bars, 30 μm (A and B) or 20 μm (C). White arrows in panel C indicate chains and aggregates of E. faecalis. (D) Sizes of E. coli aggregates in monoculture or in coculture with E. faecalis. Means of at least four independent replicates are shown; error bars indicate standard deviations. *P* values for the differences between single- and double-species biofilms were calculated using Mann-Whitney tests. **, *P* < 0.005; ns, not significant.

To further understand the underlying mechanisms, we monitored the early stages of biofilm formation for single- and dual-species cultures using fluorescence microscopy. In accordance with previous work (24), E. coli cells rapidly formed small and relatively unstable cell aggregates at the surface of the well, with the number and size reaching 8 ± 2 aggregates/1,000 μm^2^ and 80 ± 20 μm^2^, respectively, during the first hour of incubation (Fig. 3A and D). These aggregates grew slowly during the first 3 h and eventually merged to form larger structures, with ∼5 ± 1 aggregates/1,000 μm^2^ and an average size of 200 ± 20 μm^2^.

In the mixed cultures, E. faecalis cells could be observed as chains of single cells or aggregates after 1 h (Fig. 3B and C). These aggregates also seemed to incorporate E. coli cells (Fig. 3B and C), which dramatically increased the growth of E. coli aggregates. Already after 2 h, the aggregates of E. coli cells coinoculated with E. faecalis were on average 3 times larger than aggregates in E. coli-only cultures (Fig. 3A, B, and D).

Such apparent recruitment indicated that E. faecalis aggregates might chemotactically attract E. coli cells. Since AI-2 was identified previously as the autoaggregation-mediating chemotactic signal in E. coli (23, 24, 33), we hypothesized that it might also promote chemotaxis-mediated coaggregation of different species. Indeed, E. faecalis cells are known to secrete AI-2 in the exponential phase of growth (34), and we confirmed that the strain used in this study was an active producer of AI-2 (Fig. S2).

**FIG 4:**
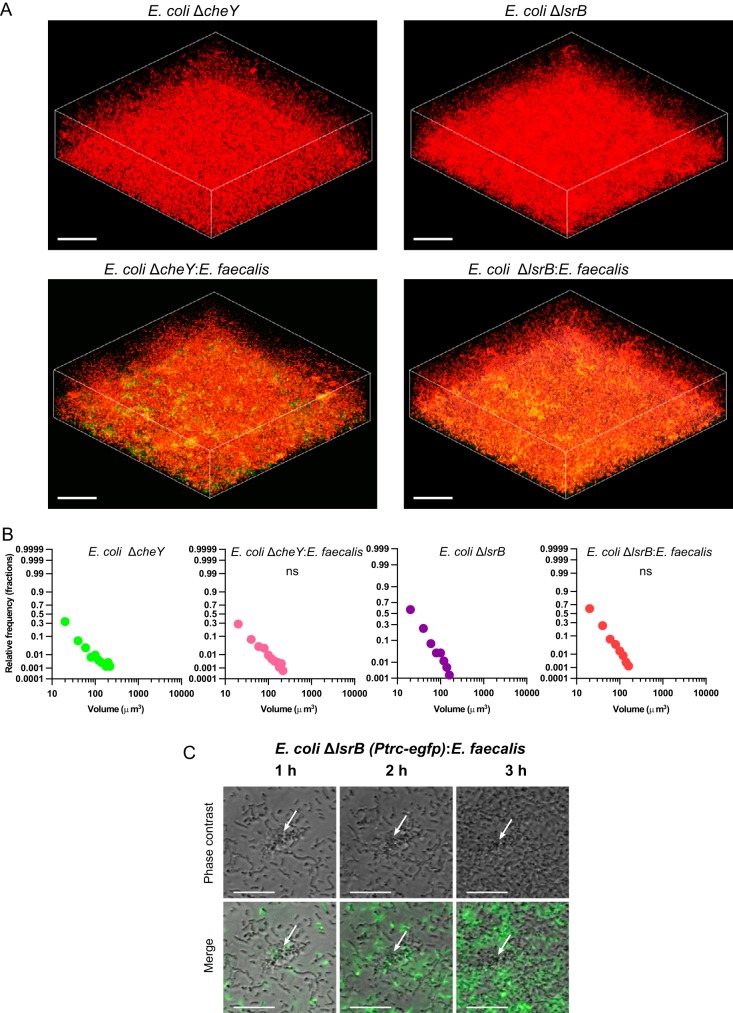
Dependence of coaggregation and mixed biofilm formation on AI-2 chemotaxis. (A) Confocal laser scanning microscopy of static biofilms of E. coli Δ*cheY* and Δ*lsrB* (expressing mCherry) grown in monoculture or mixed with E. faecalis (expressing EGFP), initially inoculated at a 1:1 ratio. Scale bars, 40 μm. (B) Distribution of microcolony volumes in the biofilms. The *P* values for the differences between single- and double-species biofilms were calculated using unpaired *t* tests (the data distribution was confirmed to be normal). ns, not significant. (C) Time-lapse fluorescence microscopy of E. coli Δ*lsrB* (expressing EGFP) grown with E. faecalis (unlabeled). The white arrows indicate an aggregate of E. faecalis.

Consistent with the role of AI-2 chemotaxis in the observed coaggregation, a non-AI-2-producing mutant of E. coli (Δ*luxS*) did not form larger microcolonies in E. coli-E. faecalis mixed biofilms (Fig. S3). This suggests that secretion of AI-2 by E. coli is required for aggregation, although interpretation of this phenotype is complicated by the known pleiotropic nature of the *luxS* deletion, which also affects motility (13, 24). More conclusively, deletion of either the key chemotaxis protein CheY, which generally abolishes chemotaxis, or a periplasmic protein (LsrB) that mediates AI-2 signaling to the chemotaxis system (23) also abolished coaggregation and enhanced formation of mixed biofilms (Fig. 4; also see Fig. S5A and B in the supplemental material). These phenotypes strongly support our hypothesis that AI-2 chemotaxis is essential for the observed enhancement of aggregation. Furthermore, coaggregation required the self-interacting E. coli adhesin Ag43 (Fig. S4 and S5C), indicating that E. faecalis aggregates do not interact directly with E. coli cells but rather serve as local sources of AI-2 that attract E. coli and initiate its autoaggregation.

### Cocultivation with E. faecalis promotes AI-2 signaling in E. coli.

In E. coli, the *lsr* operon, which includes *lsrB*, is positively regulated by AI-2. As a consequence, the operon is repressed at low cell density but becomes activated in the mid-exponential to late exponential phase, when the concentration of extracellular AI-2 becomes sufficiently high to relieve the repression. This leads in turn to enhanced AI-2 internalization and depletion from the medium, as the *lsr* operon encodes a high-affinity AI-2 importer (13).

**FIG 5:**
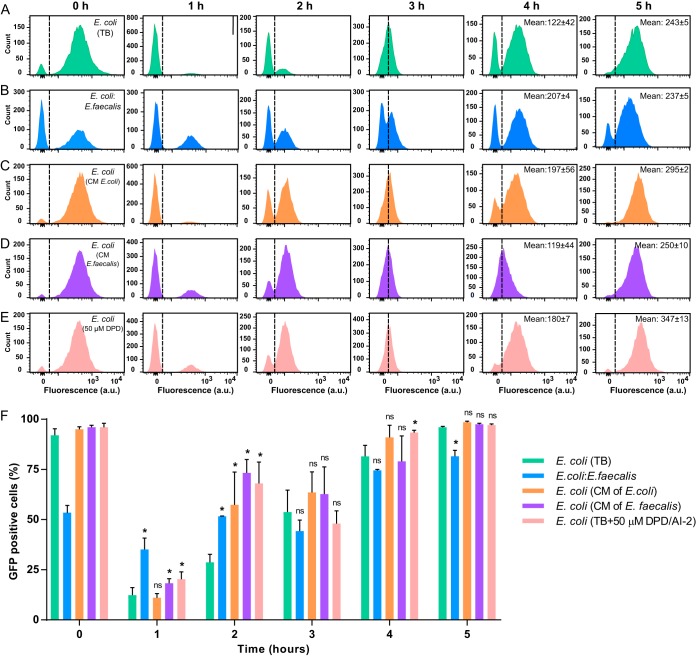
Dependence of P*lsr-egfp* activity on growth stage and AI-2 signaling. The activity of the *lsr* operon was measured using flow cytometry. (A to E) E. coli cells carrying the P*lsr-egfp* reporter plasmid pVS1723 grown in TB alone (A) or with E. faecalis at a 1:1 ratio (B), grown in conditioned medium (CM) from E. coli (C) or E. faecalis (D), or grown in TB supplemented with 50 μM synthetic DPD/AI-2 (E). Dashed lines distinguish GFP-positive (induced E. coli) and GFP-negative (uninduced E. coli, as well as unlabeled E. faecalis in panel B) subpopulations. Note that, since E. coli constitutes only 50% of the population at 0 h in panel B, the overall fraction of GFP-positive bacteria appears lower than for E. coli monocultures. (F) Percentage of GFP-positive cells in each population. Means of four independent replicates are shown; error bars indicate standard deviations. *P* values for the difference from E. coli biofilms grown in TB were calculated using Mann-Whitney tests. *, *P* < 0.05; ns, not significant.

The LsrB-dependent growth of E. coli aggregates in mixtures with E. faecalis already during the early exponential phase thus seemed surprising, as the population density in the initial stages of biofilm growth should normally be too low to allow induction of the *lsr* operon. Indeed, upon dilution of the overnight culture in fresh medium to an OD_600_ of 0.03, *lsr* operon activity was rapidly inhibited in most E. coli cells during the first hour of monoculture incubation (Fig. 5A and F). The subsequent growth resulted in gradual induction of expression, with 96% of the population expressing the *lsr* operon after 5 h.

In contrast, only about one-third of E. coli cells coinoculated with E. faecalis switched off the AI-2 system upon reinoculation (Fig. 5B and F). Thus, LsrB expression is indeed maintained in a large fraction of E. coli cells in early mixed biofilms. This effect of E. coli-E. faecalis cocultivation on the activity of the AI-2 QS system of E. coli was not contact dependent, since it was also observed in cells lacking Ag43 (Fig. S6). Such increased (compared to E. coli monocultures) expression of the *lsr* operon during early growth was apparently correlated with the elevated levels of AI-2 in the mixed cultures (Fig. S2). Indeed, *lsr* expression at the early time points was also above the control values for E. coli grown in conditioned medium from E. faecalis (Fig. 5C and F), as well as E. coli grown in conditioned medium from E. coli or in cultures with 50 μM synthetic 4,5-dihydroxy-2,3-pentanedione (DPD)/AI-2 (Fig. 5D, E, and F). All of these findings suggest that greater *lsr* expression in E. coli-E. faecalis cocultures, compared to E. coli monocultures, is indeed due to the elevated levels of AI-2. Cocultivation of E. coli with E. faecalis or the addition of synthetic DPD/AI-2 did not, however, affect the level of E. coli *luxS* promoter activity (Fig. S7), suggesting that production of AI-2 by E. coli was not altered in the presence of E. faecalis.

Lower levels of AI-2 seem not to be the only reason for decreased expression of the *lsr* operon in the early stages of growth, as substantial decreases were observed even in the presence of E. faecalis or externally added AI-2. This is in agreement with previous reports suggesting that other factors, such as the metabolic state of the cells, also contribute to *lsr* activation (35–38).

**FIG 6:**
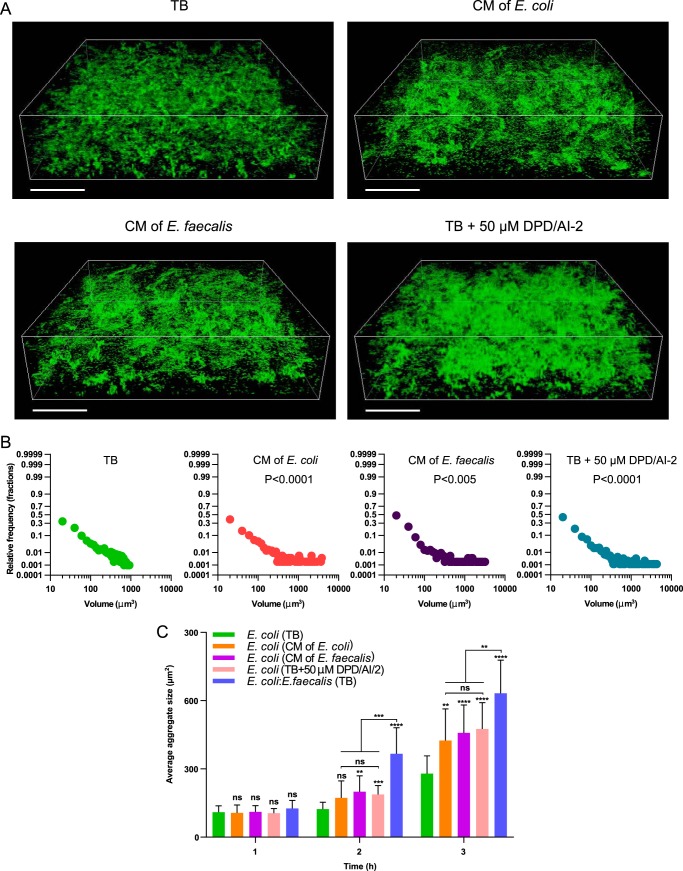
E. coli biofilm formation in conditioned media and in the presence of exogenous DPD/AI-2. (A) Confocal laser scanning microscopy of static E. coli (expressing EGFP) biofilms grown in TB, in conditioned medium (CM) from E. coli or E. faecalis, or in TB supplemented with 50 μM synthetic DPD/AI-2, as indicated. Scale bars, 40 μm. (B) Distribution of microcolony volumes in the indicated biofilms. *P* values for the differences from E. coli biofilms grown in TB were calculated using unpaired *t* tests (the data distribution was confirmed to be normal). (C) Aggregate sizes (assayed as in Fig. 2) of E. coli cells grown in TB (in monoculture or in coculture with E. faecalis), in conditioned medium from E. coli or E. faecalis, or in TB supplemented with 50 μM synthetic DPD/AI-2, as indicated. Means of at least three independent replicates are shown; error bars indicate standard deviations. *P* values for the differences from E. coli biofilms grown in TB or between indicated cultures were calculated using Mann-Whitney tests. ****, *P* < 0.0001; ***, *P* < 0.0002; **, *P* < 0.005; ns, not significant.

Further supporting the connection between *lsr* induction and increased aggregation of E. coli, stimulation by conditioned media from E. faecalis or E. coli or by synthetic DPD/AI-2 was sufficient to enhance microcolony formation in mature E. coli biofilms (Fig. 6A and B). As neither addition of synthetic DPD/AI-2 nor cocultivation with E. faecalis affected Ag43 expression or the percentage of Ag43-producing E. coli cells (Fig. S8), interspecies AI-2 signaling promotes aggregation largely by stimulating AI-2 chemotaxis of E. coli. Nevertheless, because E. coli-E. faecalis coaggregation resulted in faster aggregate growth in the earlier stages of biofilm formation than did stimulation with AI-2 or with conditioned medium (Fig. 6C), we conclude that E. faecalis enhances E. coli biofilm formation at low cell densities both globally, by relieving *lsr* operon inhibition, and locally, by nucleating formation of E. coli aggregates.

### Formation of mixed biofilms enhances stress resistance.

**FIG 7:**
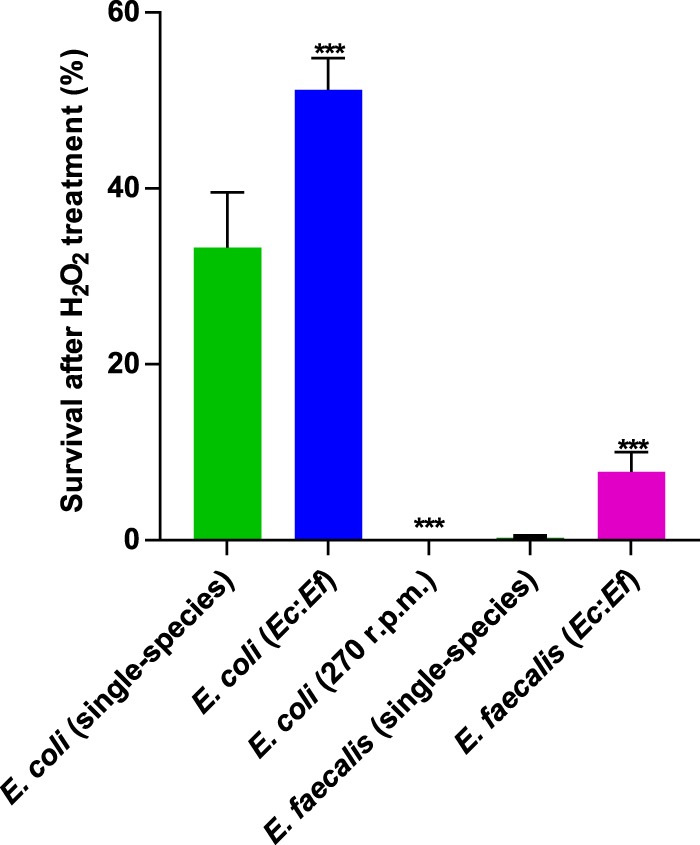
Survival of E. coli and E. faecalis in single- or double-species biofilms under oxidative stress. Single-species or mixed (*Ec*:*Ef*) biofilm cultures of E. coli and E. faecalis were exposed to 0.5% H_2_O_2_ as described in Materials and Methods. E. coli cultures incubated under nonaggregating conditions (shaking at 270 rpm) were used as controls. Means of at least five independent replicates are shown; error bars indicate standard deviations. *P* values for the differences between single- and double-species biofilms were calculated using Mann-Whitney tests. ***, *P* < 0.0002.

Aggregation and biofilm formation are generally known to enhance the stress resistance of bacteria, and it was shown previously that Ag43-mediated autoaggregation of E. coli provides protection against oxidative stress (1, 24, 39, 40). Since E. coli forms more structured biofilms with larger microcolonies when it is cocultured with E. faecalis, we hypothesized that such enhancement might promote stress resistance of E. coli, and possibly also of E. faecalis. Indeed, the survival rate of E. coli upon H_2_O_2_ treatment increased from ∼33% in a single-species biofilm to >50% in a mixed biofilm (Fig. 7). Moreover, the coaggregation in a mixed biofilm also greatly enhanced the survival rate of E. faecalis, confirming that E. faecalis cells in these biofilms are covered with E. coli microcolonies and thus are less exposed to the oxidative stress.

## DISCUSSION

In recent years, bacterial biofilms have been increasingly viewed as primarily multispecies communities, with elaborate spatial structures and complex interspecies interactions (4, 8). One of the most extensively studied forms of these interactions is a small-molecule-based mechanism of cell-cell communication known as quorum sensing (QS) (9). Because bacteria in biofilms are packed into dense aggregates, it seems obvious that QS must be relevant in natural communities. However, although there is clear evidence that QS plays distinct roles in biofilm formation by individual species, including the initial attachment phase and biofilm maturation and dispersal (41), the importance of QS in multispecies biofilms remains largely unexplored (4, 14, 16, 42, 43).

In this respect, the QS signaling mediated by AI-2 is an attractive candidate for interspecies communication, since AI-2 production and sensing is widespread among various taxonomic groups of bacteria (13, 44, 45). Indeed, the influence of AI-2 on multispecies oral biofilms (14–16, 46) and community composition in the mouse gut (20) was demonstrated, although the details of the underlying regulation remain unknown. Here we provide direct evidence that AI-2 signaling between different species can enhance biofilm formation, and we further characterize the mechanism of this enhancement.

Recent work showed that AI-2 plays a major role in E. coli biofilm formation, by mediating chemotaxis toward growing cell aggregates (24, 25). During Ag43-dependent autoaggregation, initial E. coli aggregates formed by random cell collisions secrete AI-2, which attracts other planktonic cells. In this study, we demonstrate that E. coli can also use AI-2 chemotaxis for coaggregation with E. faecalis, resulting in enhanced E. coli microcolony formation and subsequent biofilm formation in a mixed community. One apparent benefit of such coaggregation is to enable an individual species (in this case, E. coli) to aggregate at lower cell density than in monoculture, and we indeed observed that formation of E. coli aggregates occurred already during the first hours of growth in cocultures. Moreover, the formation of mixed aggregates also promoted stress resistance of both species, which could be explained by the formation of larger E. coli aggregates and the protection of E. faecalis cells incorporated in those aggregates.

**FIG 8:**
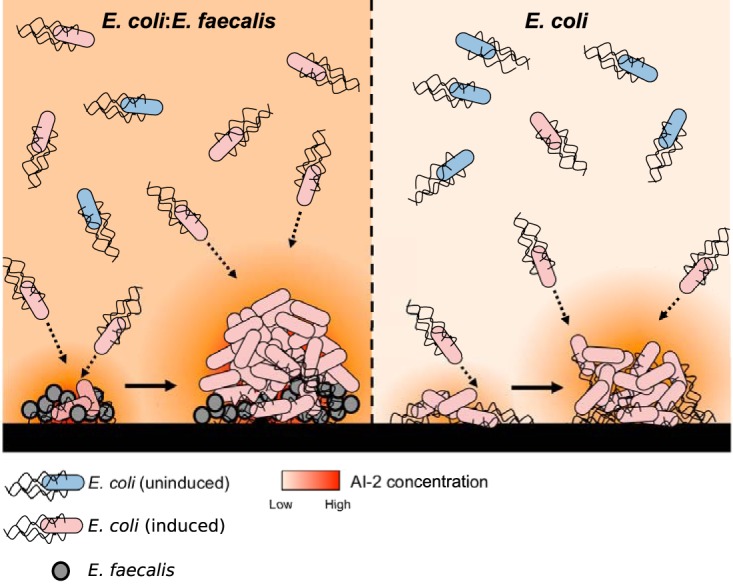
Proposed model of a static dual-species biofilm, in comparison to a single-species E. coli biofilm. E. faecalis is an active AI-2 producer, and its aggregates attract E. coli cells expressing LsrB. Cocultivation of E. coli with E. faecalis in static systems results in higher levels of extracellular AI-2, which helps E. coli cells to maintain *lsr* operon expression at low cell densities and to coaggregate effectively with E. faecalis, which creates nucleation zones for subsequent enhanced aggregate growth and biofilm formation. These coaggregates of E. coli and E. faecalis are more resistant to stress.

Another key factor in this AI-2 dependent enhancement of collective behavior in mixed cultures is sustained induction of the *E. coli lsr* operon by E. faecalis. This induction is important, because AI-2 chemotaxis requires LsrB protein, which is also a part of the cell-density-dependent Lsr system for AI-2 internalization and degradation (13, 23). As a consequence, in E. coli monocultures, AI-2-mediated autoaggregation emerges only as a population enters the mid-exponential to late exponential growth phase and the AI-2 concentration in the medium is high enough to cause derepression of the *lsr* operon (24), In contrast, greater Lsr expression in the mixed cocultures, apparently due to the additional AI-2 that is secreted by E. faecalis, enables autoaggregation of E. coli already in early stages of growth. Consistently, both sustained induction of the *lsr* operon and enhanced autoaggregation and biofilm formation could also be achieved with the addition of exogenous DPD/AI-2 to E. coli monocultures. Since induction of the *lsr* operon is the only known effect of AI-2 on gene expression in E. coli, we conclude that this induction is indeed the major cause of enhanced biofilm formation in the cocultures. However, the most prominent enhancement was observed when both factors, i.e., LsrB induction and nucleation zones provided by E. faecalis, were present (Fig. 8).

Besides providing clear evidence for the importance of cell-cell communication for the formation of mixed biofilms, our work also resolves two apparent paradoxes, namely, (i) why E. coli uses AI-2, an interspecies QS molecule, for autoaggregation and (ii) how such autoaggregation can occur in the human intestine, where E. coli constitutes a minority population and is unlikely to reach cell densities high enough either to activate the AI-2 QS system or to aggregate on its own. The use of AI-2 produced by groups of E. faecalis, another inhabitant of the human intestine (26, 47), and most likely by groups of other bacteria in the gut (17–19), as an aggregation signal could help E. coli to overcome such density limitations. More generally, we hypothesize that the strategy of chemotaxis-driven coaggregation might be common in mixed microbial communities. Particularly for minor species within the community, coaggregation might provide important means to reach local densities that are sufficient for collective behaviors such as QS induction or biofilm formation.

## MATERIALS AND METHODS

### Bacterial strains and culture conditions.

The strains and plasmids used in this study are listed in Table 1. E. coli W3110 (RpoS^+^) and E. faecalis ATCC 29212 were grown in liquid tryptone broth (TB) (10 g tryptone and 5 g NaCl per liter), supplemented with antibiotics when necessary.

**TABLE 1:** Strains and plasmids used in this study

Strain or plasmid	Relevant genotype or phenotype^*a*^	Reference or source
Strains		
E. coli W3110 (RpoS^+^)	W3110 derivative with functional RpoS, *rpoS396(Am)*	55
Enterococcus faecalis ATCC 29212	Wild-type strain isolated from urine	Leibniz Institute DSMZ German Collection of Microorganisms and Cell Cultures (Braunschweig, Germany)
VS823	W3110 Δ*luxS*; Km^s^	24
VS824	W3110 Δ*flu*; Km^s^	24
VS825	W3110 Δ*lsrB*; Km^s^	24
VS695	W3110 Δ*cheY*; Km^s^	24
CHNL13B	MC4100 *flu*::*flu*(0–48 bp)-T7RNApol; Cam^r^	48
LeoL194	W3110 *flu*::*flu*(0–48 bp)-T7RNApol; Cam^r^	This study
Plasmids		
pTrc99a	Expression vector; pBR ori, pTrc promoter, IPTG inducible; Amp^r^	56
pUA66	Expression vector; SC101 ori, GFPmut2 under control of promoter of interest; Km^r^	50
pBSU100	Expression vector; pUC ori, pAmβ1 ori, promoterless *egfp*; Spc^r^	51
pVS1515	*egfp* in pTrc99A, IPTG inducible; Amp^r^	24
pOB2	*mCherry* in pTrc99A, IPTG inducible; Amp^r^	31
pLeoL7	P*rplL-egfp* in pBSU100; Spc^r^	This study
pLeoL8	P*luxS-egfp* in pUA66; Km^r^	This study
pVS1723	P*lsr-egfp* in pUA66; Km^r^	24
pHL32	P(T7)-*gfpmut3.1*; Km^r^	48

a Km^s^, kanamycin sensitive; Km^r^, kanamycin resistant; Cam^r^, chloramphenicol resistant; Amp^r^, ampicillin resistant; Spc^r^, spectinomycin resistant.

A genomic reporter construct for assessing Ag43 (*flu*) expression (48) was introduced into the E. coli W3110 genome via P1 transduction (49). This construct enables amplification of the signal from the *flu* promoter by replacing its coding sequence with the T7 RNA polymerase gene. The resulting strain, LeoL194, was then transformed with the pHL32 plasmid carrying the *gfpmut3.1* gene controlled by the T7 polymerase promoter (48). A plasmid-based reporter for *luxS* expression was constructed by cloning the sequence containing a 926-nucleotide region upstream and a 23-nucleotide region downstream of the *luxS* start codon from the E. coli W3110 genome into the pUA66 plasmid carrying the promoterless *egfp* gene (50).

### Fluorescent labeling of E. faecalis.

The reporter plasmid pLeoL7 was constructed in order to visualize E. faecalis cells in mixed biofilms. The −350 to +17 nucleotide region of the E. faecalis constitutively expressed *rplL* gene was cloned into the pBSU100 shuttle vector carrying a promoterless copy of *egfp* (51). Transformation of E. faecalis cells was performed as described elsewhere (52). The fluorescence signal from this plasmid was low and only allowed imaging using confocal laser scanning microscopy.

### Confocal laser scanning microscopy of static biofilms.

For two-color labeling, overnight cultures of E. coli carrying plasmid pOB2 carrying *mCherry* under the control of the isopropyl-β-d-thiogalactopyranoside (IPTG)-inducible *trc* promoter and E. faecalis carrying *egfp* under the control of the constitutively expressed *rplL* promoter were diluted in TB containing 5 μM IPTG, to a final OD_600_ of 0.03. For single-color labeling of E. coli, plasmid pVS1515 carrying *egfp* under the control of the *trc* promoter was used. For dual-species biofilm cultivation, the same amounts of E. coli and E. faecalis cells were coinoculated, resulting in a final OD_600_ of 0.06; 400 μl of each sample was cultivated for 24 h at 37°C in 8-well glass-bottom slides (μ-Slide, 8-well glass bottom; ibidi). The biofilms were visualized using a Zeiss LSM-800 microscope (apochromat 40× objective), and z-stack images were acquired and analyzed using ZEN Black software (Zeiss).

Three-dimensional structures of mature E. coli biofilms (green fluorescent protein [GFP] positive) were quantified using the 3D objects counter plugin for ImageJ (53). The plugin allows quantification of the volumes of patches formed by connected fluorescent cells.

### Flow cytometry.

Levels of *lsr* operon induction were assayed using a plasmid-based reporter containing the 217-nucleotide region upstream of the *lsrA* gene fused to *egfp* (24). Samples were prepared as described above and diluted 1:20 in tethering buffer (10 mM KH_2_PO_4_, 100 μM EDTA, 1 μM l-methionine, and 10 mM lactic acid [pH 7.0]), and fluorescence was measured every hour with a BD LSRFortessa SORP cell analyzer (BD Biosciences, Germany).

For quantification of AI-2 levels in supernatants, a non-AI-2-producing biosensor strain was used, and the quantification was performed as described previously (24). Where indicated, synthetic DPD (obtained from Rita Ventura, ITQB, Oeiras, Portugal) (54) solution was added to the samples; it is referred to as DPD/AI-2 because of its spontaneous conversion into AI-2. Cell-free conditioned medium was prepared by filtration, through 0.2-μm filters, of supernatants collected from statically grown cultures (1 h at 37°C).

Biofilm growth rates were determined by counting the GFP-positive (E. coli) and GFP-negative (E. faecalis) cells in the samples (1:400 dilution in tethering buffer). The biofilms were disrupted by pipetting and subsequent vortex-mixing of the samples. During cell counting, the flow rate was set to 6 μl/min, with an acquisition time of 10 s.

### Fluorescence microscopy.

The initial stages of biofilm formation in 8-well glass-bottom slides were assessed with a Nikon Eclipse Ti-E microscope equipped with an Andor Zyla 4.2 sCMOS camera (Andor Technology Ltd., UK), a plan apochromat 40× objective, and a filter set for GFP. Images were acquired every hour using NIS-Elements AR software (Nikon) and were analyzed using the particle analysis tool (ImageJ) to determine the size of aggregates (the detection threshold was set to 5 μm^2^).

### Hydrogen peroxide treatment.

Cell survival upon treatment with H_2_O_2_ was determined as described previously (24, 40). Briefly, E. coli, E. faecalis, or E. coli-E. faecalis static biofilms were grown in 400 μl TB for 24 h at 37°C. Subsequently, 5.7 μl of 35% H_2_O_2_ was added to each sample, resulting in a final H_2_O_2_ concentration of 0.5%, and samples were incubated for 15 min at room temperature. CFU for each sample before and after treatment were counted by plating serial dilutions of cultures. For mixed biofilms, CFU of E. coli and E. faecalis could be easily counted separately based on differences in their colony morphology on LB plates. The survival rate was determined as (posttreatment CFU/initial CFU) × 100%.

## Supplementary Material

Supplemental material

## References

[1] Hall-Stoodley L, Costerton J, Stoodley P 2004 Bacterial biofilms: from the natural environment to infectious diseases. Nat Rev Microbiol 2:95–108. doi:10.1038/nrmicro821. 15040259

[2] Bjarnsholt T, Alhede M, Alhede M, Eickhardt-Sørensen SR, Moser C, Kühl M, Jensen PØ, Høiby N 2013 The in vivo biofilm. Trends Microbiol 21:466–474. doi:10.1016/j.tim.2013.06.002. 23827084

[3] Davey ME, O'Toole GA 2000 Microbial biofilms: from ecology to molecular genetics. Microbiol Mol Biol Rev 64:847–867. doi:10.1128/MMBR.64.4.847-867.2000. 11104821 PMC99016

[4] Burmølle M, Ren D, Bjarnsholt T, Sørensen SJ 2014 Interactions in multispecies biofilms: do they actually matter? Trends Microbiol 22:84–91. doi:10.1016/j.tim.2013.12.004. 24440178

[5] Liu W, Røder HL, Madsen JS, Bjarnsholt T, Sørensen SJ, Burmølle M 2016 Interspecific bacterial interactions are reflected in multispecies biofilm spatial organization. Front Microbiol 7:1366. doi:10.3389/fmicb.2016.01366. 27630624 PMC5005372

[6] Tolker-Nielsen T, Molin S 2000 Spatial organization of microbial biofilm communities. Microb Ecol 40:75–84. 11029076 10.1007/s002480000057

[7] Nadell CD, Drescher K, Foster KR 2016 Spatial structure, cooperation and competition in biofilms. Nat Rev Microbiol 14:589–600. doi:10.1038/nrmicro.2016.84. 27452230

[8] Elias S, Banin E 2012 Multi-species biofilms: living with friendly neighbors. FEMS Microbiol Rev 36:990–1004. doi:10.1111/j.1574-6976.2012.00325.x. 22229800

[9] Bassler BL 2002 Small talk: cell-to-cell communication in bacteria. Cell 109:421–424. doi:10.1016/S0092-8674(02)00749-3. 12086599

[10] Miller MB, Bassler BL 2001 Quorum sensing in bacteria. Annu Rev Microbiol 55:165–199. doi:10.1146/annurev.micro.55.1.165. 11544353

[11] Surette MG, Miller MB, Bassler BL 1999 Quorum sensing in *Escherichia coli*, *Salmonella typhimurium*, and *Vibrio harveyi*: a new family of genes responsible for autoinducer production. Proc Natl Acad Sci U S A 96:1639–1644. doi:10.1073/pnas.96.4.1639. 9990077 PMC15544

[12] Bassler BL, Greenberg EP, Stevens AM 1997 Cross-species induction of luminescence in the quorum-sensing bacterium *Vibrio harveyi*. J Bacteriol 179:4043–4045. doi:10.1128/jb.179.12.4043-4045.1997. 9190823 PMC179216

[13] Pereira CS, Thompson JA, Xavier KB 2013 AI-2-mediated signalling in bacteria. FEMS Microbiol Rev 37:156–181. doi:10.1111/j.1574-6976.2012.00345.x. 22712853

[14] McNab R, Ford SK, El-Sabaeny A, Barbieri B, Cook GS, Lamont RJ 2003 LuxS-based signaling in *Streptococcus gordonii*: autoinducer 2 controls carbohydrate metabolism and biofilm formation with *Porphyromonas gingivalis*. J Bacteriol 185:274–284. doi:10.1128/JB.185.1.274-284.2003. 12486064 PMC141908

[15] Cuadra-Saenz G, Rao DL, Underwood AJ, Belapure SA, Campagna SR, Sun Z, Tammariello S, Rickard AH 2012 Autoinducer-2 influences interactions amongst pioneer colonizing streptococci in oral biofilms. Microbiology 158:1783–1795. doi:10.1099/mic.0.057182-0. 22493304 PMC3542140

[16] Rickard AH, Palmer RJ, Blehert DS, Campagna SR, Semmelhack MF, Egland PG, Bassler BL, Kolenbrander PE 2006 Autoinducer 2: a concentration-dependent signal for mutualistic bacterial biofilm growth. Mol Microbiol 60:1446–1456. doi:10.1111/j.1365-2958.2006.05202.x. 16796680

[17] Antunes LCM, Ferreira LQ, Ferreira EO, Miranda KR, Avelar KE, Domingues RMCP, Ferreira MCS 2005 *Bacteroides* species produce *Vibrio harveyi* autoinducer 2-related molecules. Anaerobe 11:295–301. doi:10.1016/j.anaerobe.2005.03.003. 16701587

[18] Lukáš F, Gorenc G, Kopečný J 2008 Detection of possible AI-2-mediated quorum sensing system in commensal intestinal bacteria. Folia Microbiol (Praha) 53:221–224. doi:10.1007/s12223-008-0030-1. 18661296

[19] Schauder S, Shokat K, Surette MG, Bassler BL 2001 The LuxS family of bacterial autoinducers: biosynthesis of a novel quorum-sensing signal molecule. Mol Microbiol 41:463–476. doi:10.1046/j.1365-2958.2001.02532.x. 11489131

[20] Thompson JA, Oliveira RA, Ubeda C, Xavier KB, Djukovic A 2015 Manipulation of the quorum sensing signal AI-2 affects the antibiotic-treated gut microbiota. Cell Rep 10:1861–1871. doi:10.1016/j.celrep.2015.02.049. 25801025

[21] Gonzalez Barrios AF, Zuo R, Hashimoto Y, Yang L, Bentley WE, Wood TK 2006 Autoinducer 2 controls biofilm formation in *Escherichia coli* through a novel motility quorum-sensing regulator (MqsR, B3022). J Bacteriol 188:305–316. doi:10.1128/JB.188.1.305-316.2006. 16352847 PMC1317603

[22] Bansal T, Jesudhasan P, Pillai S, Wood TK, Jayaraman A 2008 Temporal regulation of enterohemorrhagic *Escherichia coli* virulence mediated by autoinducer-2. Appl Microbiol Biotechnol 78:811–819. doi:10.1007/s00253-008-1359-8. 18256823

[23] Hegde M, Englert DL, Schrock S, Cohn WB, Vogt C, Wood TK, Manson MD, Jayaraman A 2011 Chemotaxis to the quorum-sensing signal AI-2 requires the Tsr chemoreceptor and the periplasmic LsrB AI-2-binding protein. J Bacteriol 193:768–773. doi:10.1128/JB.01196-10. 21097621 PMC3021223

[24] Laganenka L, Colin R, Sourjik V 2016 Chemotaxis towards autoinducer 2 mediates autoaggregation in *Escherichia coli*. Nat Commun 7:12984. doi:10.1038/ncomms12984. 27687245 PMC5056481

[25] Jani S, Seely AL, Peabody VGL, Jayaraman A, Manson MD 2017 Chemotaxis to self-generated AI-2 promotes biofilm formation in *Escherichia coli*. Microbiology 163:1778–1790. doi:10.1099/mic.0.000567. 29125461

[26] Lebreton F, Willems RJL, Gilmore MS 2014 *Enterococcus* diversity, origins in nature, and gut colonization. Gilmore MS, Clewell DB, Ike Y, Shankar N (ed), *Enterococci*: from commensals to leading causes of drug resistant infection. Massachusetts Eye and Ear Infirmary, Boston, MA. 24649513

[27] Flores-Mireles AL, Walker JN, Caparon M, Hultgren SJ 2015 Urinary tract infections: epidemiology, mechanisms of infection and treatment options. Nat Rev Microbiol 13:269–284. doi:10.1038/nrmicro3432. 25853778 PMC4457377

[28] Keogh D, Tay WH, Ho YY, Dale JL, Chen S, Umashankar S, Williams RBH, Chen SL, Dunny GM, Kline KA 2016 Enterococcal metabolite cues facilitate interspecies niche modulation and polymicrobial infection. Cell Host Microbe 20:493–503. doi:10.1016/j.chom.2016.09.004. 27736645 PMC5076562

[29] van der Woude MW, Henderson IR 2008 Regulation and function of Ag43 (*flu*). Annu Rev Microbiol 62:153–169. doi:10.1146/annurev.micro.62.081307.162938. 18785838

[30] Danese PN, Pratt LA, Dove SL, Kolter R 2000 The outer membrane protein, antigen 43, mediates cell-to-cell interactions within *Escherichia coli* biofilms. Mol Microbiol 37:424–432. doi:10.1046/j.1365-2958.2000.02008.x. 10931336

[31] Besharova O, Suchanek VM, Hartmann R, Drescher K, Sourjik V 2016 Diversification of gene expression during formation of static submerged biofilms by *Escherichia coli*. Front Microbiol 7:1568. doi:10.3389/fmicb.2016.01568. 27761132 PMC5050211

[32] Vidal O, Longin R, Prigent-Combaret C, Dorel C, Hooreman M, Lejeune P 1998 Isolation of an *Escherichia coli* K-12 mutant strain able to form biofilms on inert surfaces: involvement of a new *ompR* allele that increases curli expression. J Bacteriol 180:2442–2449. 9573197 10.1128/jb.180.9.2442-2449.1998 PMC107187

[33] Englert DL, Manson MD, Jayaraman A 2009 Flow-based microfluidic device for quantifying bacterial chemotaxis in stable, competing gradients. Appl Environ Microbiol 75:4557–4564. doi:10.1128/AEM.02952-08. 19411425 PMC2704821

[34] Shao C, Shang W, Yang Z, Sun Z, Li Y, Guo J, Wang X, Zou D, Wang S, Lei H, Cui Q, Yin Z, Li X, Wei X, Liu W, He X, Jiang Z, Du S, Liao X, Huang L, Wang Y, Yuan J 2012 LuxS-dependent AI-2 regulates versatile functions in *Enterococcus faecalis* V583. J Proteome Res 11:4465–4475. doi:10.1021/pr3002244. 22856334

[35] Surette MG, Bassler BL 1998 Quorum sensing in *Escherichia coli* and *Salmonella typhimurium*. Microbiology 95:7046–7050. 10.1073/pnas.95.12.7046 PMC22733 9618536

[36] Wang L, Hashimoto Y, Tsao CY, Valdes JJ, Bentley WE 2005 Cyclic AMP (cAMP) and cAMP receptor protein influence both synthesis and uptake of extracellular autoinducer 2 in *Escherichia coli*. J Bacteriol 187:2066–2076. doi:10.1128/JB.187.6.2066-2076.2005. 15743955 PMC1064054

[37] Xavier KB, Bassler BL 2005 Regulation of uptake and processing of the quorum-sensing autoinducer AI-2 in *Escherichia coli*. J Bacteriol 187:238–248. doi:10.1128/JB.187.1.238-248.2005. 15601708 PMC538819

[38] Mitra A, Herren CD, Patel IR, Coleman A, Mukhopadhyay S, Simmons R 2016 Integration of AI-2 based cell-cell signaling with metabolic cues in *Escherichia coli*. PLoS One 11:e0157532. doi:10.1371/journal.pone.0157532. 27362507 PMC4928848

[39] Klebensberger J, Lautenschlager K, Bressler D, Wingender J, Philipp B 2007 Detergent-induced cell aggregation in subpopulations of *Pseudomonas aeruginosa* as a preadaptive survival strategy. Environ Microbiol 9:2247–2259. doi:10.1111/j.1462-2920.2007.01339.x. 17686022

[40] Schembri MA, Hjerrild L, Gjermansen M, Klemm P 2003 Differential expression of the *Escherichia coli* autoaggregation factor antigen 43. J Bacteriol 185:2236–2242. doi:10.1128/JB.185.7.2236-2242.2003. 12644494 PMC151503

[41] Parsek MR, Greenberg EP 2005 Sociomicrobiology: the connections between quorum sensing and biofilms. Trends Microbiol 13:27–33. doi:10.1016/j.tim.2004.11.007. 15639629

[42] Heras B, Totsika M, Peters KM, Paxman JJ, Gee CL, Jarrott RJ, Perugini MA, Whitten AE, Schembri MA 2014 The antigen 43 structure reveals a molecular Velcro-like mechanism of autotransporter-mediated bacterial clumping. Proc Natl Acad Sci U S A 111:457–462. doi:10.1073/pnas.1311592111. 24335802 PMC3890832

[43] Li Y-H, Tian X 2012 Quorum sensing and bacterial social interactions in biofilms. Sensors 12:2519–2538. doi:10.3390/s120302519. 22736963 PMC3376616

[44] Chen X, Schauder S, Potier N, Van Dorsselaer A, Pelczer I, Bassler BL, Hughson FM 2002 Structural identification of a bacterial quorum-sensing signal containing boron. Nature 415:545–549. doi:10.1038/415545a. 11823863

[45] Miller ST, Xavier KB, Campagna SR, Taga ME, Semmelhack MF, Bassler BL, Hughson FM 2004 *Salmonella typhimurium* recognizes a chemically distinct form of the bacterial quorum-sensing signal AI-2. Mol Cell 15:677–687. doi:10.1016/j.molcel.2004.07.020. 15350213

[46] Huang R, Li M, Gregory RL 2011 Bacterial interactions in dental biofilm. Virulence 2:435–444. doi:10.4161/viru.2.5.16140. 21778817 PMC3322631

[47] Eckburg PB, Bik EM, Bernstein CN, Purdom E, Dethlefsen L, Sargent M, Gill SR, Nelson KE, Relman DA 2005 Diversity of the human intestinal microbial flora. Science 308:1635–1638. doi:10.1126/science.1110591. 15831718 PMC1395357

[48] Lim HN, van Oudenaarden A 2007 A multistep epigenetic switch enables the stable inheritance of DNA methylation states. Nat Genet 39:269–275. doi:10.1038/ng1956. 17220888

[49] Miller JH 1972 Experiments in molecular genetics. Cold Spring Harbor Laboratory Press, Cold Spring Harbor, NY.

[50] Zaslaver A, Bren A, Ronen M, Itzkovitz S, Kikoin I, Shavit S, Liebermeister W, Surette MG, Alon U 2006 A comprehensive library of fluorescent transcriptional reporters for *Escherichia coli*. Nat Methods 3:623–628. doi:10.1038/nmeth895. 16862137

[51] Aymanns S, Mauerer S, van Zandbergen G, Wolz C, Spellerberg B 2011 High-level fluorescence labeling of Gram-positive pathogens. PLoS One 6:e19822. doi:10.1371/journal.pone.0019822. 21731607 PMC3120757

[52] Friesenegger A, Fiedler S, Devriese LA, Wirth R 1991 Genetic transformation of various species of *Enterococcus* by electroporation. FEMS Microbiol Lett 63:323–327. doi:10.1111/j.1574-6968.1991.tb04549.x. 1905659

[53] Bolte S, Cordielières FP 2006 A guided tour into subcellular colocalization analysis in light microscopy. J Microsc 224:213–232. doi:10.1111/j.1365-2818.2006.01706.x. 17210054

[54] Ascenso OS, Marques JC, Santos AR, Xavier KB, Ventura MR, Maycock CD 2011 An efficient synthesis of the precursor of AI-2, the signalling molecule for inter-species quorum sensing. Bioorg Med Chem 19:1236–1241. doi:10.1016/j.bmc.2010.12.036. 21216605

[55] Serra DO, Richter AM, Klauck G, Mika F, Hengge R 2013 Microanatomy at cellular resolution and spatial order of physiological differentiation in a bacterial biofilm. mBio 4:e00103-13. doi:10.1128/mBio.00103-13. 23512962 PMC3604763

[56] Amann E, Ochs B, Abel K-J 1988 Tightly regulated *tac* promoter vectors useful for the expression of unfused and fused proteins in *Escherichia coli*. Gene 69:301–315. doi:10.1016/0378-1119(88)90440-4. 3069586

