# Supplementary material for: Autoinducer 2-Dependent Escherichia coli Biofilm Formation Is Enhanced in a Dual-Species Coculture
Source: Appl Environ Microbiol. 2018 Feb 14;84(5):e02638-17. doi: 10.1128/AEM.02638-17 (PMC5812939; doi:10.1128/AEM.02638-17)
Supplement: Supplemental material [file supp_84_5_e02638-17__index.html]

Supplemental material 

# Autoinducer 2-Dependent Escherichia coli Biofilm Formation Is Enhanced in a Dual-Species Coculture

## Supplemental material

- Supplemental file 1 -

  Static biofilms of *E. coli* in single- or dual-species culture (Fig. S1 and S5); extracellular AI-2 levels (Fig. S2); *E. coli*-*E. faecalis* coaggregation requirement of AI-2 production (Fig. S3) and Ag43 (Fig. S4); contact independence of the effect of *E. faecalis* on induction of the *lsr* operon in *E. coli* cells (Fig. S6); lack of effect of *E. faecalis* or exogenous DPD/AI-2 on *E. coli luxS* promoter (Fig. S7) or *agn43* expression (Fig. S8).

  PDF, 1.4M
