# Supplementary material for: Autoinducer 2-Dependent Escherichia coli Biofilm Formation Is Enhanced in a Dual-Species Coculture
Source: Appl Environ Microbiol. 2018 Feb 14;84(5):e02638-17. doi: 10.1128/AEM.02638-17 (PMC5812939; doi:10.1128/AEM.02638-17)
Supplement: Supplemental material [file AEM.02638-17_zam005188341s1.pdf]

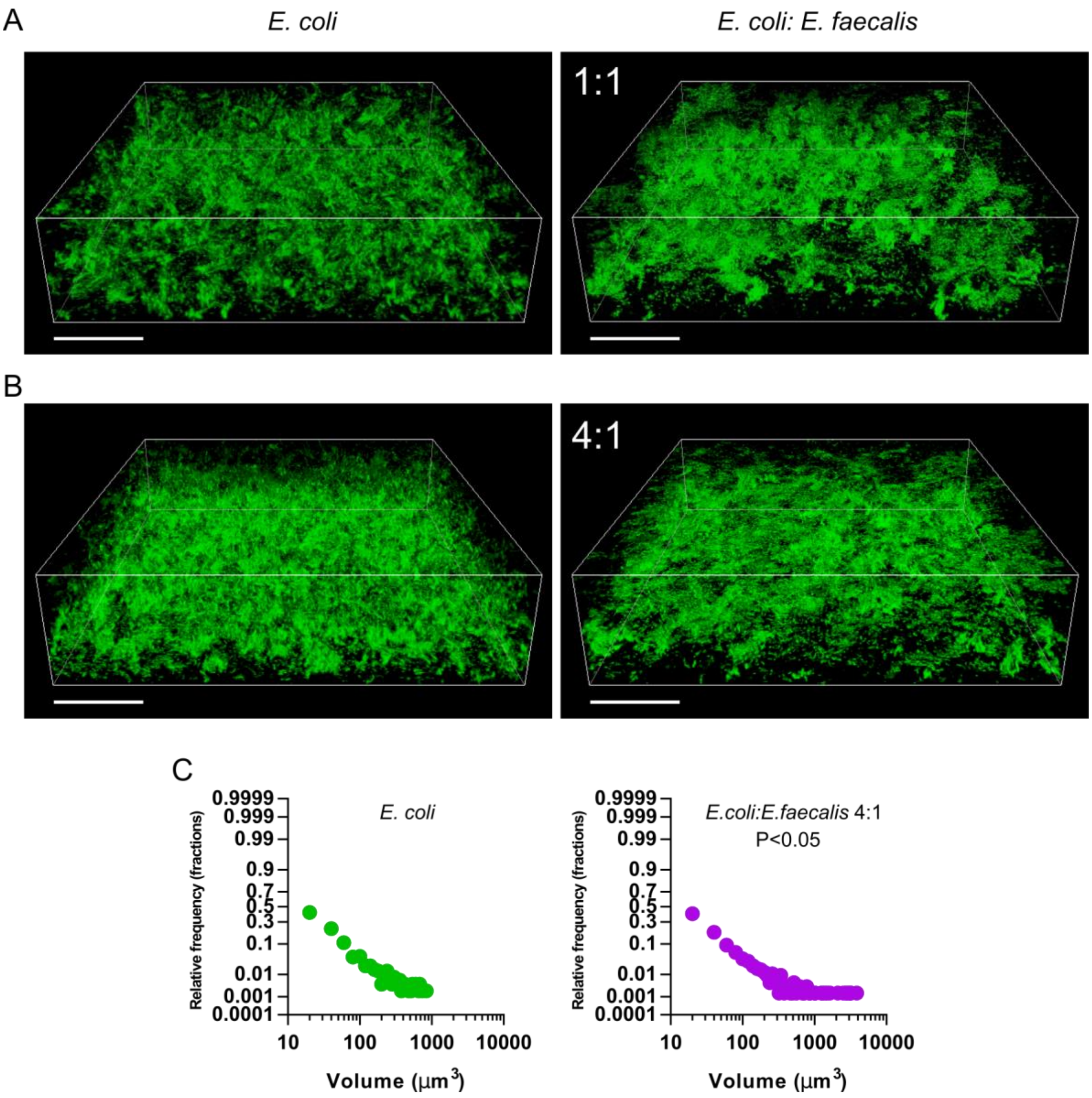

Figure S1. Static biofilms of *E. coli* in single- or dual-species culture. (A) Confocal laser scanning microscopy of static *E. coli* (GFP^+) biofilms grown in monoculture (left panel) or mixed with *E. faecalis* (right panel). Inoculation ratio was 1:1. Scale bars, 40 µm. **(B)** Confocal laser scanning microscopy of static *E. coli* (expressing GFP) biofilms grown in monoculture (left panel) or mixed with unlabeled *E. faecalis* (right panel). Inoculation ratio was 4:1. Scale bars, 40 µm. **(C)** Distribution of microcolony volumes in the biofilms. *P* value for the difference between single- and double-species biofilms was calculated using unpaired *t*-test (data distribution was confirmed to be normal).

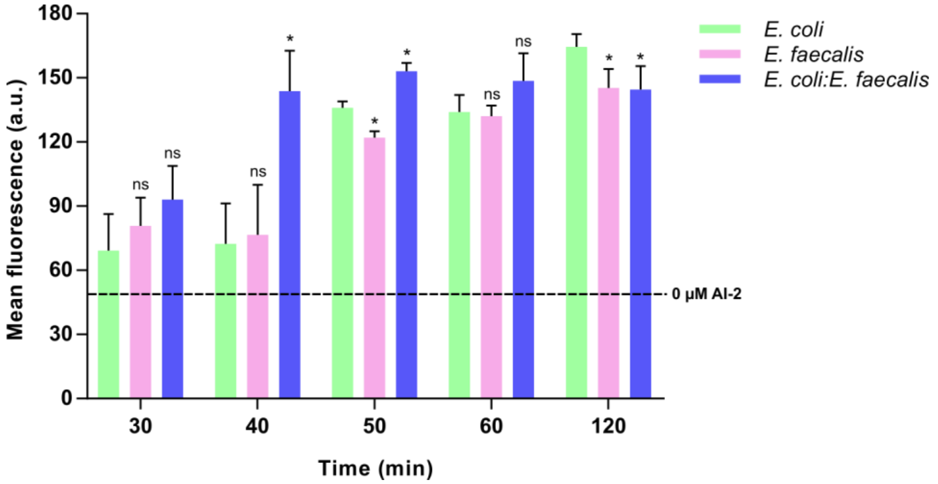

Figure S2. Extracellular AI-2 levels in *E. coli*, *E. faecalis* and *E. coli*:*E. faecalis* cultures as assessed by P*lsr-gfp* activity of the biosensor defective in AI-2 production (see Materials and Methods). Dashed line represents P*lsr-gfp* activity in absence of AI-2. Means of three independent replicates are shown; error bars represent standard deviation. *P* values were calculated using Mann-Whitney test (*$P<0.05$).

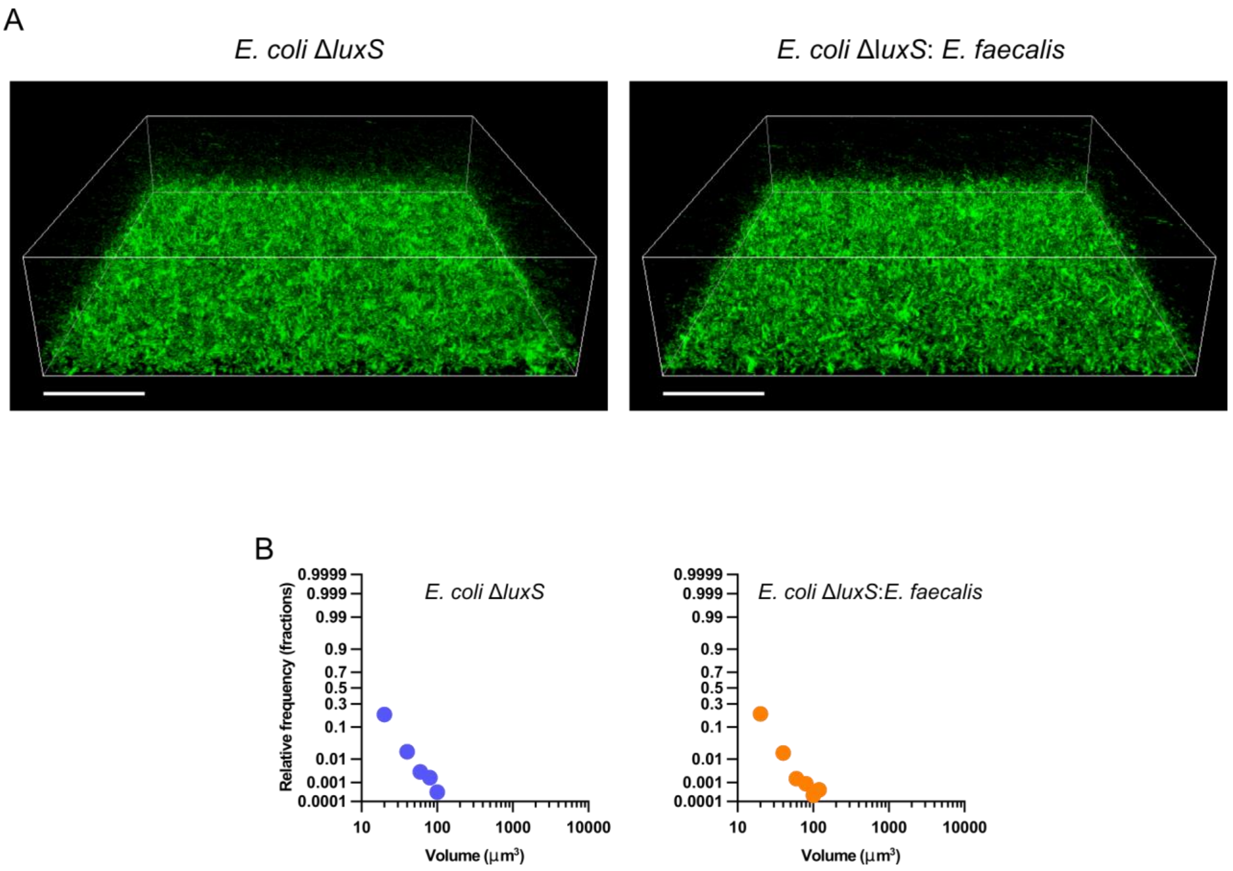

Figure S3. The *E. coli*:*E. faecalis* co-aggregation requires AI-2 production. Confocal laser scanning microscopy of static *E. coli* Δ*luxS* (expressing GFP) biofilms grown alone or in presence of unlabeled *E. faecalis* (initial inoculation ratio 1:1). Scale bars, 40 µm. **(B)** Distribution of microcolony volumes in static single- and double-species biofilms of *E. coli*. *P* value for the difference between single- and double-species biofilms calculated using unpaired *t*-test was 0.55 (data distribution was confirmed to be normal).

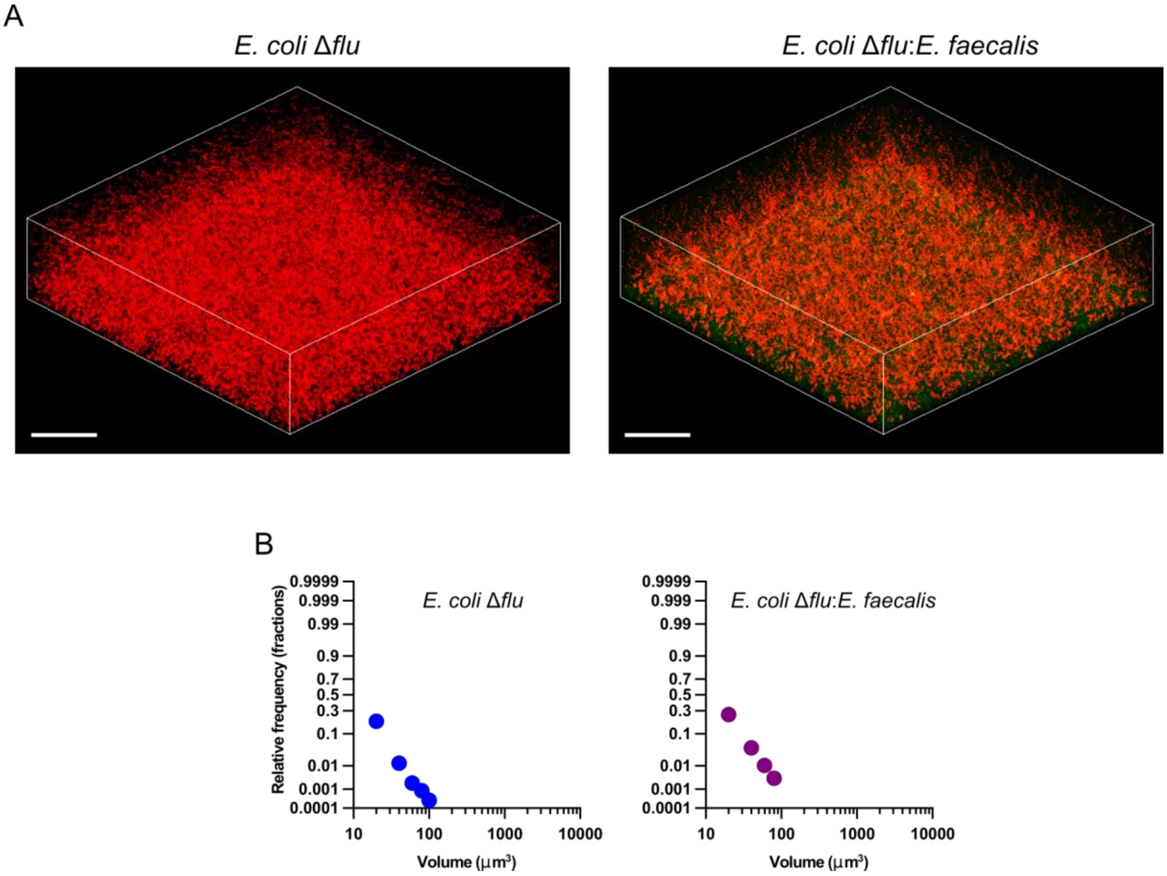

Figure S4. The *E. coli*:*E. faecalis* co-aggregation requires Ag43. Confocal laser scanning microscopy of static *E. coli* Δ*flu* (expressing mCherry) biofilms grown alone or in presence of *E. faecalis* (expressing GFP, initial inoculation ratio 1:1). Scale bars, 40 µm. **(B)** Distribution of microcolony volumes in static single- and double-species biofilms of *E. coli*. *P* value for the difference between single- and double-species biofilms calculated using unpaired *t*-test was 0.001 (data distribution was confirmed to be normal).

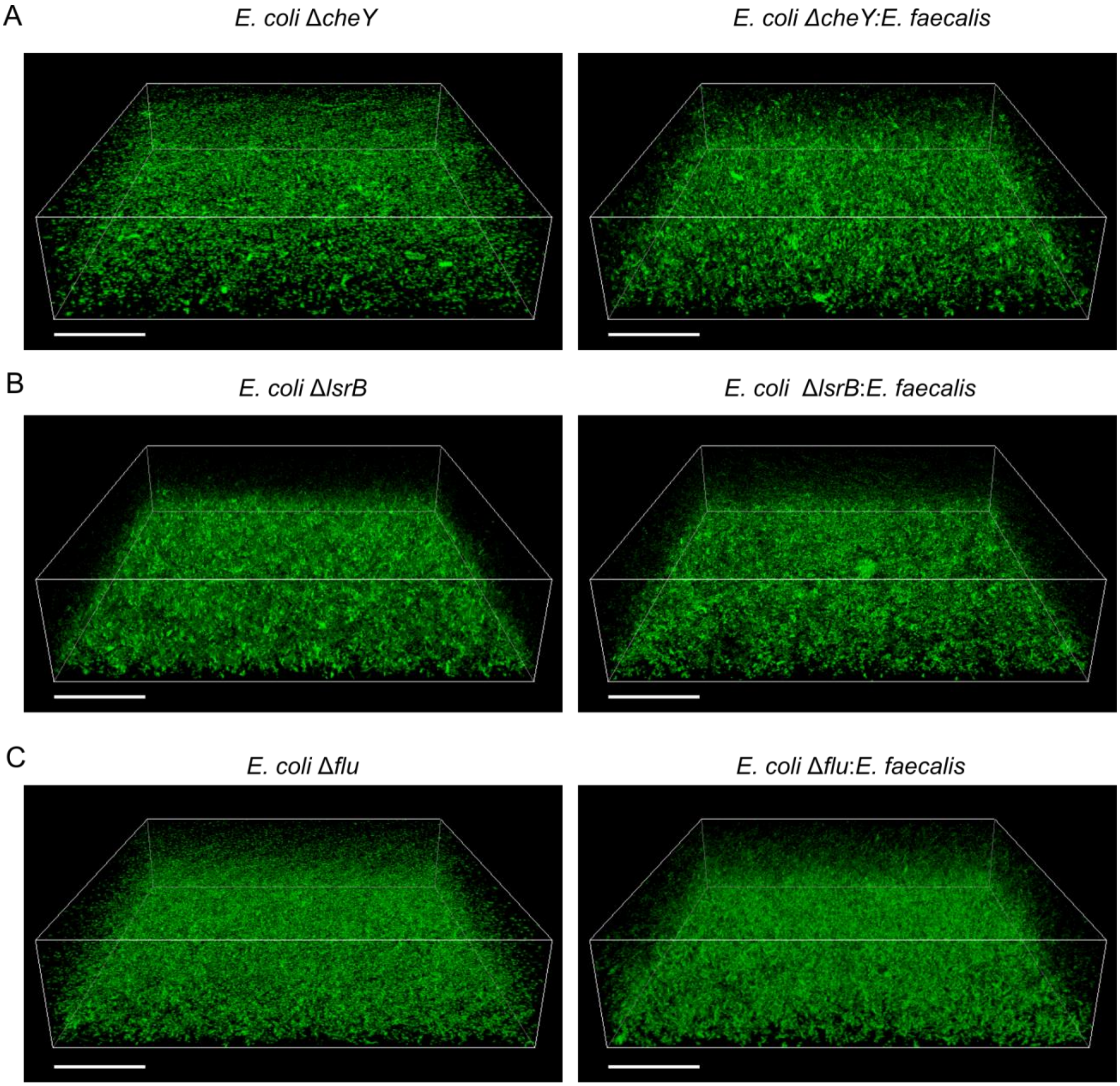

Figure S5. Static biofilms of *E. coli* in single- or dual-species culture. Confocal laser scanning microscopy of static *E. coli* (GFP^{+}) biofilms grown in monoculture (left panel) or mixed with *E. faecalis* (right panel). Inoculation ratio was 1:1. Scale bars, 40 µm.

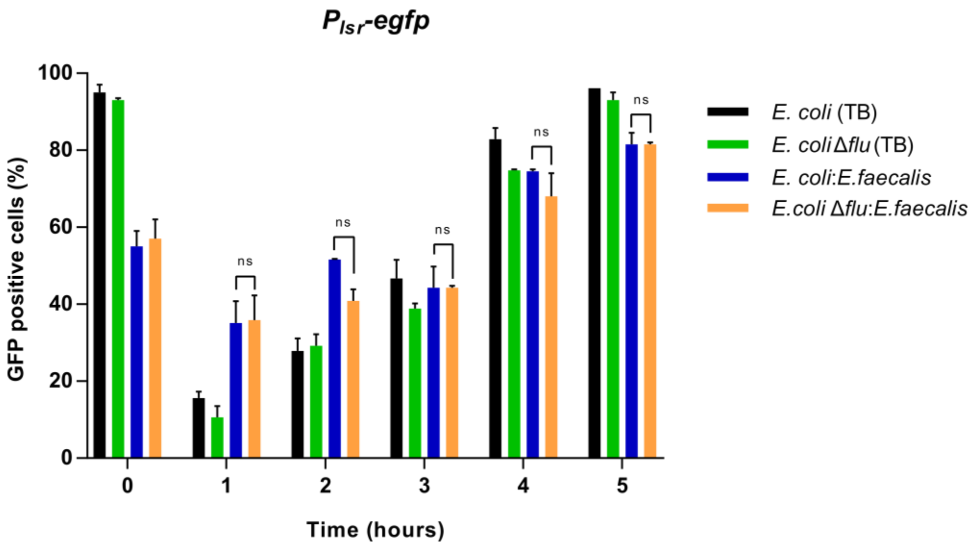

Figure S6. The effect of *E. faecalis* on induction of *lsr* operon in *E. coli* cells is contact-independent. The bars represent percentage of GFP^+ (induced) cells in each population. Note that the lower initial fraction of GFP+ cells in mixed culture is because *E. coli* initially constitutes only 50% of the total population. Means of four independent replicates are shown; error bars indicate standard deviation. *P* values for the difference from *E. coli* culture grown in TB were calculated using Mann-Whitney test (ns – not significant).

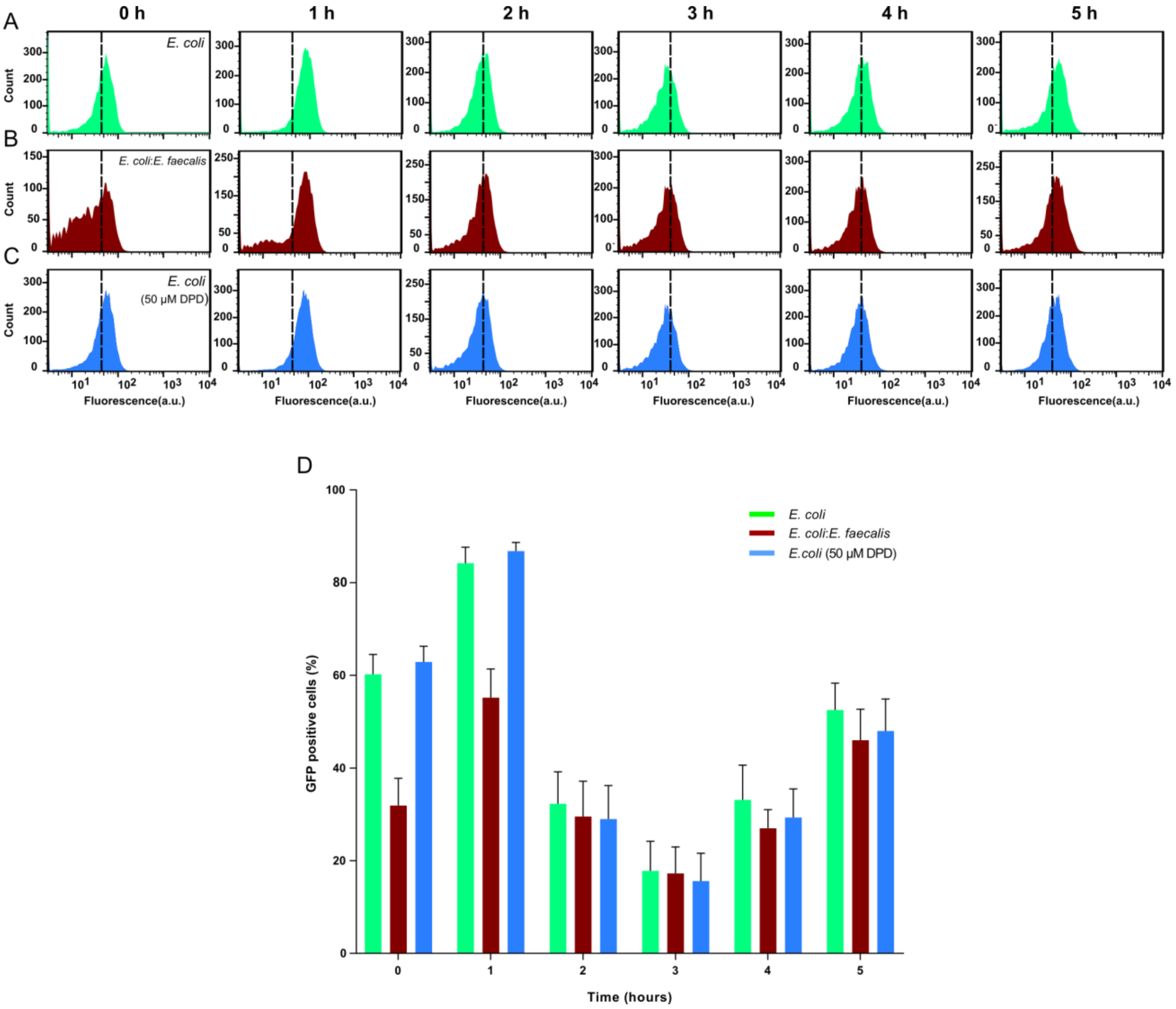

Figure S7. No effect of *E. faecalis* or exogenous DPD/AI-2 on *E. coli luxS* promoter. Activity of the *luxS* promoter was measured using flow cytometry. **(A-C)** *E. coli* cells carrying P*luxS*-*gfp* reporter plasmid pLeoL8 were grown in TB alone (A) or with *E. faecalis* at 1:1 ratio (B), or in TB supplemented with 50 µM synthetic DPD/AI-2 (C). Dashed line distinguishes GFP^+ (induced *E. coli*) and GFP^- (uninduced *E. coli* as well as unlabeled *E. faecalis* in B) subpopulations. Note that since *E. coli* comprises only 50% of population at 0 h in (B), its peak appears lower than that of *E. coli* grown alone. **(D)** The bars represent percentage of GFP^+ (induced) cells in each

population. Means of at least six independent replicates are shown; error bars indicate standard deviation.

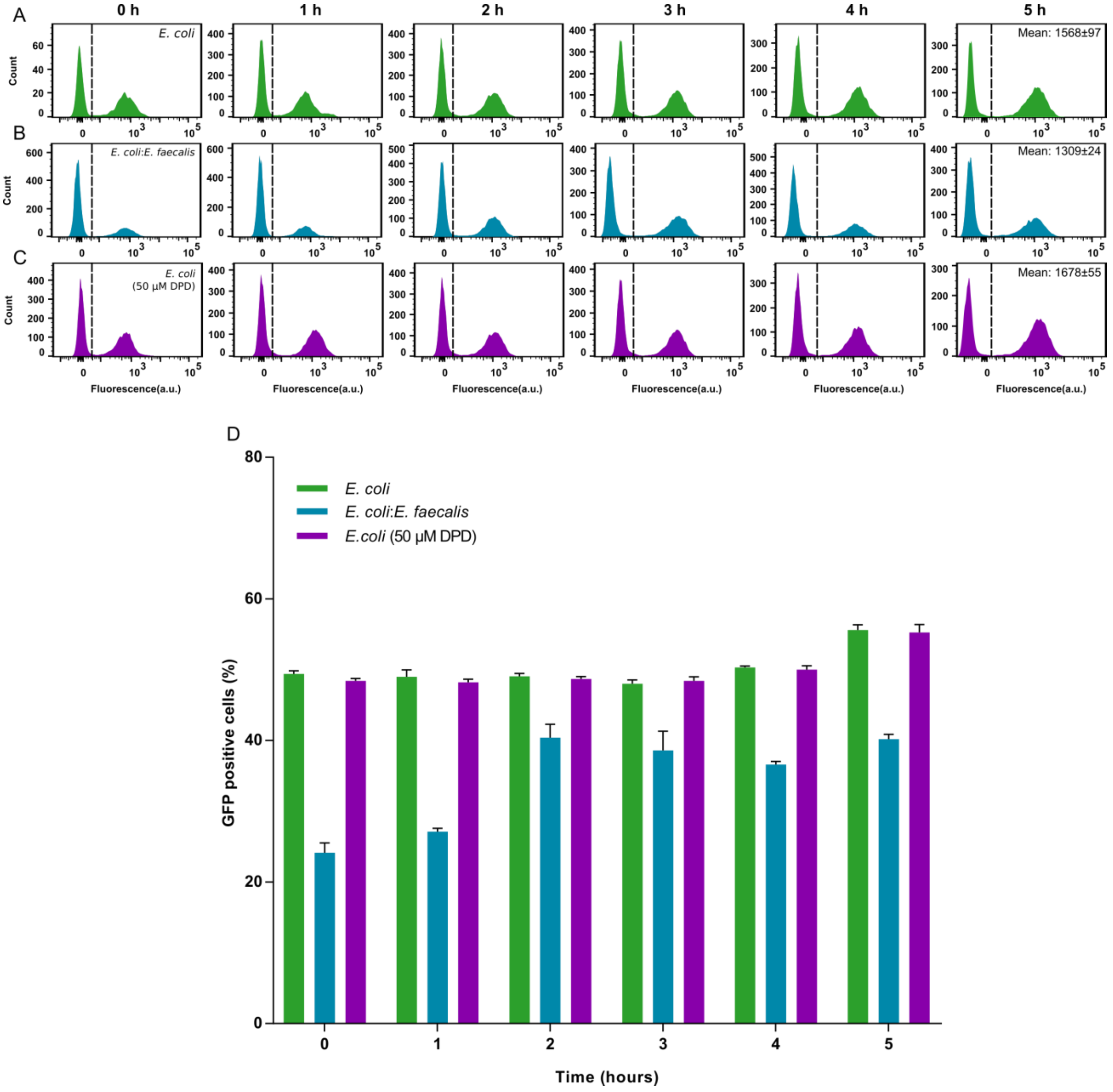

Figure S8. No effect of *E. faecalis* or exogenous DPD/AI-2 on *E. coli agn43* expression. Activity of the *agn43 (flu)* promoter was measured using flow cytometry. **(A-C)** *E. coli* LeoL194 cells carrying *agn43* reporter construct were grown in TB alone (A) or with *E. faecalis* at 1:1 ratio (B), or in TB supplemented with 50 µM synthetic DPD/AI-2 (C). Dashed line distinguishes

GFP$^+$ (induced *E. coli*) and GFP$^-$ (uninduced *E. coli* as well as unlabeled *E. faecalis* in B) subpopulations. Note that since *E. coli* comprises only 50% of population at 0 h in (B), its peak appears lower than that of *E. coli* grown alone. **(D)** The bars represent percentage of GFP$^+$ (induced) cells in each population. Means of at least four independent replicates are shown; error bars indicate standard deviation.
